# Retraction Note: Bacteria eat nanoprobes for aggregation-enhanced imaging and killing diverse microorganisms

**DOI:** 10.1038/s41467-026-73179-w

**Published:** 2026-05-13

**Authors:** Yunmin Yang, Binbin Chu, Jiayi Cheng, Jiali Tang, Bin Song, Houyu Wang, Yao He

**Affiliations:** Suzhou Key Laboratory of Nanotechnology and Biomedicine, Institute of Functional Nano and Soft Materials (FUNSOM), and Collaborative Innovation Center of Suzhou Nano Science and Technology, Soochow University, 215123 Suzhou, China

**Keywords:** Antimicrobials, Bacteria, Imaging techniques, Nanoparticles

Retraction to: *Nature Communications* 10.1038/s41467-022-28920-6, published online 10 March 2022

The Editor has retracted this article. Two panels in figure 1c representing different bacteria cells appear to partially overlap. An additional concern was raised regarding Figure 4c II, Left (UV+) and Right (UV-) which although are not the same image, appear to be images of the same site. The authors did not provide a sufficient response to the concerns raised. The Editor has lost confidence in the data and conclusions of this article.

Houyu Wang disagrees with this retraction. Yunmin Yang, Binbin Chu, Jiayi Cheng, Jiali Tang, Bin Song and Yao He did not respond to correspondence from the Publisher about this retraction.

